# Living with Food Hypersensitivity as an Adolescent Impairs Health Related Quality of Life Irrespective of Disease Severity: Results from a Population-Based Birth Cohort

**DOI:** 10.3390/nu13072357

**Published:** 2021-07-09

**Authors:** Marina Jonsson, Sandra Ekström, Jennifer L. P. Protudjer, Anna Bergström, Inger Kull

**Affiliations:** 1 Institute of Environmental Medicine, Karolinska Institutet, 171 77 Stockholm, Sweden; sandra.ekstrom@ki.se (S.E.); anna.bergstrom@ki.se (A.B.); 2 Centre for Occupational and Environmental Medicine, Region Stockholm, Solnavägen 4, 113 65 Stockholm, Sweden; 3 Department of Clinical Science and Education, Södersjukhuset, Karolinska Institutet, 118 83 Stockholm, Sweden; inger.kull@ki.se; 4 Department of Pediatrics and Child Health, Rady Faculty of Health Sciences, University of Manitoba, Winnipeg, MB R3A 1S1, Canada; Jennifer.Protudjer@umanitoba.ca; 5 George and Fay Yee Centre for Healthcare Innovation, Winnipeg, MB R3E 0T6, Canada; 6 The Children’s Hospital Research Institute of Manitoba, Winnipeg, MB R3E 3P4, Canada; 7 The Centre for Allergy Research, Karolinska Institutet, 171 77 Stockholm, Sweden; 8 Sachs’ Children and Youth Hospital, 118 83 Stockholm, Sweden

**Keywords:** adolescence, allergic comorbidity, food allergy, food hypersensitivity, IgE sensitization, health-related quality of life

## Abstract

Food hypersensitivity (FHS) refers to food-related symptoms, with or without concurrent Immunoglobulin E (IgE) antibodies related to food(s). It remains unclear how different FHS phenotypes affect health-related quality of life (HRQoL). We examined self-reported HRQoL (with the generic instrument EQ-5D (dimensions and a Visual Analogue Scale (VAS), range 0–100) in association with phenotypes of FHS, and food-specific symptoms among adolescents (n = 2990) from a population-based birth cohort. Overall, 22% of the adolescents had FHS. Compared to adolescents without FHS, those with FHS reported more problems in the dimensions of pain/discomfort (*p* < 0.001), and anxiety/depression (*p* = 0.007). Females with FHS reported more problems than males in these dimensions (*p* < 0.001). Different FHS phenotypes (IgE-sensitization, allergic co-morbidity, and severity of symptoms) were not associated with differences in HRQoL. EQ-VAS scores were lowest for adolescents with symptoms for wheat vs. no wheat, median 80 vs. 89, *p* = 0.04) and milk vs. no milk (median 85 vs. 90, *p* = 0.03). Physician-diagnosed lactose intolerance median EQ-VAS was 80 vs. 90, *p* = 0.03 and also associated with more problems in the dimension of anxious/depression. In conclusion, FHS is associated with lower HRQoL in adolescence, irrespective of phenotypes, but differentially affects females vs. males, and those with vs. without symptoms for milk or wheat.

## 1. Introduction

Food hypersensitivity (FHS) covers a large spectrum of food-related symptoms and can occur with or without concurrent Immunoglobulin E (IgE) antibodies to the affected food items. FHS is an increasing public health issue throughout the world and affects all age groups [1,2,3]. During childhood, FHS is common, with a noted increase in prevalence in recent decades [4,5]. Among adolescents, the prevalence is estimated at 15–25 percent [1,6,7,8,9,10,11]. Although self-reported perceptions of food-related symptoms are common among both children and adolescents, a German study showed that only one out of ten could be confirmed with IgE-related symptoms [10]. Notably, those with FHS in combination with IgE sensitization tend to have more severe disease [7,8]. Data from our population-based birth cohort (BAMSE) show that FHS often persists into adolescence, where 74% of those with FHS in combination with IgE sensitization at pre-school age also had symptoms at age 16 years, compared to 36% of those with FHS without IgE sensitization [11]. Further, adolescents with FHS in combination with IgE sensitization often have other allergic manifestations, such as asthma, rhinitis and eczema [12].

Food-related symptoms can range from mild symptoms (i.e., involving the nose, eyes, stomach and/or skin) to life-threatening, when symptoms involve respiratory and/or cardiovascular distress [13]. Those who are severely allergic to certain food(s) must avoid those foods due to the risk of anaphylaxis, a potentially fatal reaction. Whereas most individuals will not experience severe symptoms, living with FHS involves a constant risk assessment surrounding food consumption [14]. This is often described as being the most difficult when eating outside the home [15]. However, notably, 50% of anaphylactic reactions occur at home [16,17].

Adolescence marks a life stage during which individuals take greater responsibility for their FHS, yet remain largely dependent on their parents/guardians. Adolescence is also a vulnerable period, owing to physical, physiological, and social changes. These changes may be further complicated when living with FHS [18,19]. Adolescents commonly desire to be like their peers and live without limitations [20]. One widely accepted key patient-reported outcome in the fields of health and medicine is health-related quality of life (HRQoL) [21,22]. To enhance well-being among adolescents, it is important to study factors associated with HRQoL [23]. We previously reported that adolescents with asthma have a lower HRQoL compared to adolescents without asthma [24]. Previous reports from our group and from others provide further evidence that children and adolescents with FHS have low HRQoL [25,26,27,28,29]. Children with more severe FHS, asthma comorbidity or experienced anaphylaxis have lower quality of life than those with less severe disease [30,31,32]. Among younger adolescents (ages 12–13 years), different phenotypes of FHS were shown to have no further impact on HRQoL, but tended to impact on those with a current food allergy [33]. However, it remains unclear how different phenotypes and symptom severity affect HRQoL in later adolescence. Moreover, it cannot be ruled out that the report of food-related symptoms is a proxy for a personality type with more anxiety and stress. As FHS is increasing in prevalence and is thus an increasing public health concern, further knowledge is needed to understand how different FHS phenotypes, including IgE-sensitization, allergic co-morbidity, and symptom severity, affect HRQoL. As such, the primary aim of this study was to examine self-reported HRQoL in association with different FHS phenotypes amongst adolescents from a population-based birth cohort. The secondary aim was to examine how symptoms to different foods affect HRQoL amongst adolescents with FHS.

## 2. Material and Methods

### 2.1. Study Design and Population

This study is based on data from the population-based, prospective birth cohort, BAMSE, which consists of 4089 children (at baseline) born between 1994 and 1996, in predefined areas of Stockholm, Sweden [34]. Parent-reported information on environmental exposures, socioeconomic status, and family history of allergic disease was obtained when the children were approximately 2 months old. Follow-up studies were performed at regular intervals (ages 1, 2, 4, 8, 12 and 16 years). The response rate at the 16-year follow-up was 78%, with data obtained from web-based (91%) or paper (9%) questionnaires. 

From the clinical examination, sera were obtained at age 16 years and analyzed for IgE reactivity to common food allergens with fx5 ^®^ (egg, milk, codfish, wheat, peanut, soy) (Thermo Fisher/Phadia AB, Uppsala, Sweden).

### 2.2. Assessment of HRQoL

Self-reported HRQoL by adolescents was obtained with the generic instrument EQ-5D, appropriate for all ages and diverse populations [35,36,37]. The EQ-5D captures the respondent’s current health status and consists of a descriptive system investigating five dimensions of mobility, self-care, usual activities, pain/discomfort, and anxiety/depression. Within each dimension, there are three levels of severity: no problems, some problems, and extreme problems. The EQ-5D also consists of a standard visual analogue scale (EQ-VAS) with end points ranging from 0 for “worst imaginable health state”, to 100 for “best imaginable health state”. The EQ-5D was intended to capture health status at the time of the questionnaires.

### 2.3. Assessments of Food Hypersensitivity

FHS

Specific parent-reported symptoms (such as vomiting, diarrhea, eczema, urticaria, itch or swelling of the lips and/or eyelids, runny nose, and/or asthma) in response to one or more specific food(s), namely milk, egg, fish, tree nuts, peanuts, peas, soy, wheat, and/or fruit with pits/pips in the last 12 months or avoiding one or more specific food(s) due to previous reactions or results of allergy testing [11].

Specific foods

The specific foods were classified into the following mutually exclusive groups:

- Common foods (milk, egg, fish and wheat).
- Peanuts and tree nuts eanut, hazelnut, almond, walnut/pecan, cashew/pistachio andBrazil nut).

Other specific foods (shellfish, soy, sesame, apple/pear, peach/nectarine/plum/cherry, kiwi, banana, and raw carrot).

Physician-diagnosed lactose intolerance

Parent-reported physician-diagnosed lactose intolerance ever, in combination specific symptoms to milk in the last 12 months prior to the 16-year follow-up. 

Mild to moderate symptoms

Parent-reported symptoms of itchy nose, stuffy nose, runny nose, itchy eyes, nettle rash that covered less of the body, stomach pain/vomiting, swollen face, eyelids, lips or itch in mouth, throat or ears to at least one of the reported food [11].

Severe symptoms

Parent-reported symptoms of difficulty breathing asthma cough, nettle rash covering most of the body, hoarseness, indistinct speech, pronounced fatigue, decreased awareness, unconsciousness to at least one of the reported foods [11,38].

IgE sensitization

Adolescents were categorized as IgE-sensitized if they had an IgE antibody level ≥0.35 kUA/L to fx5 ^®^ (food mix of egg, milk, codfish, wheat, peanut, soy).

Allergic comorbidity

Allergic comorbidity was defined as FHS, in addition to meeting the definition of asthma and or/eczema and/or rhinitis at the 16-year follow-up. Complete definitions are presented in the Supplemental Text S1: Allergic comorbidity (FHS in combination with asthma, eczema or rhinitis).

Any adolescent-reported reactions to food.

At the 16-year follow-up, adolescents answered a single question on symptoms to foods in the last 12 months: “In the past 12 months, have you had a reaction to anything in your food”?

### 2.4. Statistics

All statistical analyses were performed with Stata Statistical Software (release 13.0, College Station, TX, USA). One-sample *t*-test with a finite population correction was used to test differences in background factors between the original cohort and the study population. Differences between groups with regard to the number of subjects with and without reported problems in the EQ-5D descriptive system were tested with a chi-square test. Between-group differences in mean and median EQ-VAS scores were tested with a two-sample *t*-test and a two-sample Wilcoxon–Mann–Whitney’s rank-sum test, respectively. A *p*-value of ≤0.05 was considered statistically significant.

Three types of regression analyses were used to examine the associations between FHS, sex, FHS phenotypes and the outcome, HRQoL. Logistic regression was used for categorical variables (the five dimensions of EQ-5D), linear regression was used for mean EQ-VAS, and quantile regression was used for median EQ-VAS. In the adjusted models, we included potential confounders which may have impacted on HRQoL in adolescence, as seen in Table S2 (parental occupation, overweight/obesity and self-reported smoking).

To minimize the risk that report of FHS could be a proxy for a personality that experiences more stress and anxiety and not true “FHS”, we linked data to the Swedish Prescribed Drug Register [39] on antianxiety drugs (Anatomical Therapeutic Chemical (ATC) N05B + R06DA), antidepressant drugs (ATC N06A) and dispensed migraine medications (ATC N02CC), the last 18 months prior to the 16-year follow-up. The above medications were classified in accordance with the ATC classification system [40].

A sensitivity analyses was performed using the adolescent-reported data on food-symptoms (in the 12 months prior to the 16-year follow-up) and EQ-5D.

The study population consisted of participants with data from the 16-year follow-up with information on symptoms of FHS, allergic diseases, and self-reported HRQoL (n = 2990). A sub-population consisted of adolescents who participated in a clinical examination including blood sampling on allergen-specific IgE (n = 2477).

The study was approved by the Ethics Committee of the Karolinska Institutet, Stockholm, Sweden (application number KI 2010/1474-31/3).

## 3. Results

### 3.1. Background Characteristics and Self-Reported HRQoL in the Study Population

The background characteristics of the study population (n = 2990) were similar to those of the original cohort except for a statistically significant higher socioeconomic status (assessed by parental occupation) and a lower rate of parental tobacco smoking at baseline (Table S1: Distribution of baseline characteristics of the entire cohort and the study population).

In the study population, 1.07% (n = 32) of the adolescents reported some or extreme problems in the EQ-5D dimension mobility, in selfcare (0.74%; n = 22), activity (3.18%; n = 76), pain/discomfort (18.96%; n = 569) and in anxious/depression (28.85%; n = 683). The reported median EQ-VAS was 90. Information about HRQoL in relation to background characteristics showed that females had significantly lower HRQoL compared to males (*p* < 0.001) in the dimensions—pain/discomfort and anxiety/depression; and in the EQ-VAS scale. This was also observed among males and females without FHS. Parental socioeconomic status was associated with lower HRQoL, with blue collar workers reporting more problems in the dimension pain or discomfort (*p* = 0.02) than white collar workers. Compared to adolescent non-smokers, adolescents who reported smoking also reported lower HRQoL in usual activities, anxiety/depression, median EQ-VAS (all *p* < 0.001), and in the dimension pain/discomfort (*p* = 0.03). Adolescents who were overweight/obese had lower HRQoL in the dimension mobility (*p* = 0.005) and in pain/discomfort (*p* = 0.006), (Table S2: Distribution of EQ 5D in the study-population in relation to selected background characteristics).

### 3.2. Background Characteristics and Self-Reported HRQoL in Relation to FHS

In total, 22% (n = 650) of the study population fulfilled the definition of FHS at the 16-year follow-up. The prevalence of FHS was higher among females compared to males (24% vs. 19%, *p* = 0.004). When comparing those with vs. without FHS in relation to prevalence of dispensed antianxiety drugs, and/or antidepressant drugs and/or migraine drugs, there were no significant differences (i.e., 3.3% vs. 3.5%, *p* = 0.71).

The majority of adolescents with or without FHS reported no problems in the EQ-5D dimensions (Table 1). Yet, adolescents with FHS reported significantly more problems in the dimensions usual activities (4.92% vs. 3.25%, *p* = 0.04), pain or discomfort (24.15% vs. 17.52%, *p* < 0.001), and anxiety or depression (26.77% vs. 21.75%, *p* = 0.007) compared to those without FHS (Table 1). A small but statistically significant difference was also seen in the EQ-VAS scale (median of 89 vs. 90 *p* = 0.001). In the adjusted analyses, a significant association was presented in the dimension pain/discomfort (OR_adj_ 1.37, 95% CI (1.08–1.72)) anxiety/depression (OR_adj_ 1.24 95% CI (0.99–1.55)) and in mean EQ-VAS (β-coefficient-1.4, 95% CI (−2.64, −0.19). No significant association was seen in the dimension activity or in median EQ-VAS, (Table 1).

Among those with FHS, females reported significantly more problems in the dimensions of pain or discomfort (29.76% vs. 17.07%, *p* < 0.001) and anxiety or depression (31.96% vs. 20.21% *p* < 0.001), compared to males (Table 2). Females also had a lower EQ-VAS score compared to males (median 85 vs. 90, *p*-value < 0.001). To assess the association between sex and HRQoL in those with FHS, we performed regression analyses adjusting for potential confounders (parental occupation, overweight/obesity and self-reported smoking). Female sex was associated with a significantly lower HRQoL in the dimensions of pain/discomfort (OR_adj_ 0.55, 95% CI (0.33–0.77), and anxiety/depression (OR_adj_ 0.55, 95% CI (0.37–0.82) and in the median EQ-VAS (OR_adj_ 2.0, 95% CI (1.24–6.76)) (Table 2).

### 3.3. Self-Reported HRQoL in Relation to Phenotypes of FHS

Among those with FHS, 74% (n = 438) of adolescents reported mild/moderate symptoms and 26% (n = 155) reported severe symptoms, with no differences in HRQoL identified between the two groups (Table 3). Allergic comorbidity was common among adolescents with FHS; 60% had asthma, eczema or rhinitis, again with no differences in HRQoL between the two groups (Table S3: Distribution of food hypersensitivity with and without allergic comorbidity, asthma and/or eczema and/or, rhinitis).

In the sub-population (n = 2477) with information on IgE sensitization (with the cut of ≥0.35 kUA/L) to food allergens, 29% (n = 164) of those with FHS (n = 561) were IgE-sensitized to common food allergens (milk, egg, fish, soy, wheat, and peanut). However, no difference in HRQoL was seen in relation to IgE sensitization (Table 4), even after adjusting for potential confounders (results not shown). To capture the occurrence of symptoms, we also used ≥0.7 kUA/L as a threshold for IgE sensitization in relation to HRQoL. No significance differences were shown here.

### 3.4. Self-Reported HRQoL in Relation to Symptoms on Specific Foods among Adolescents with FHS

**Figure 1:**
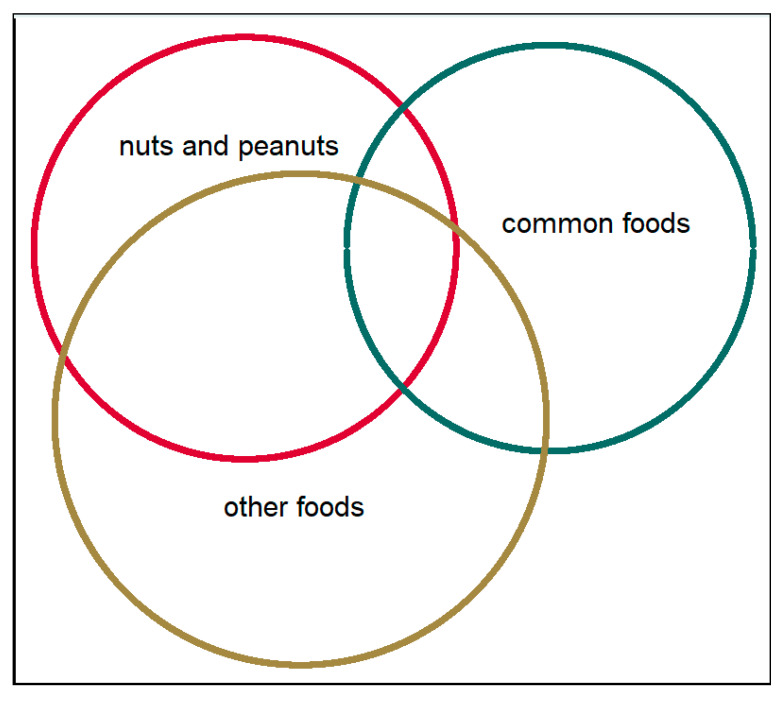
The distribution of symptoms of different food items among adolescents with food hypersensitivity. Common foods: milk, egg, fish and wheat. Nuts and peanuts: peanut, hazelnut, almond, walnut/pecan, cashew/pistachio, Brazil nuts. Other foods: Shellfish, soy, sesame seed, apple/pear, peach/nectarine/plum/cherry, kiwi, banana and raw carrot.

There was an overlap between symptoms in relation to different food groups in those with FHS (Figure 1). Symptoms after consumption of common foods (milk, egg, wheat, and fish) were reported by 31% (milk n = 203, egg n = 28, fish n = 19, wheat n = 30). Among those with symptoms to common foods, the majority (81%) had mild-to-moderate symptoms, whereas 19% reported severe symptoms.

Symptoms after consumption of peanuts and nuts were reported by 41% (n = 270), with comparable distributions of mild-to-moderate symptoms and severe symptoms (51% vs. 49%,). Symptoms after consumption of other foods were reported by 56% (n = 366), of whom 73% reported mild to-moderate symptoms and 27% reported severe symptoms.

With consideration of symptoms to common foods, those with symptoms reported a lower EQ-VAS than those without symptoms (median 85 vs. 90, *p* = 0.04; Table S4: Distribution of EQ-5D in relation to food hypersensitivity and symptoms on common foods, nuts/peanuts and other specific foods) No statistically significant differences in EQ-5D were found between those with symptoms vs. without symptoms, to other specific foods, or to nuts and peanuts.

**Figure 2:**
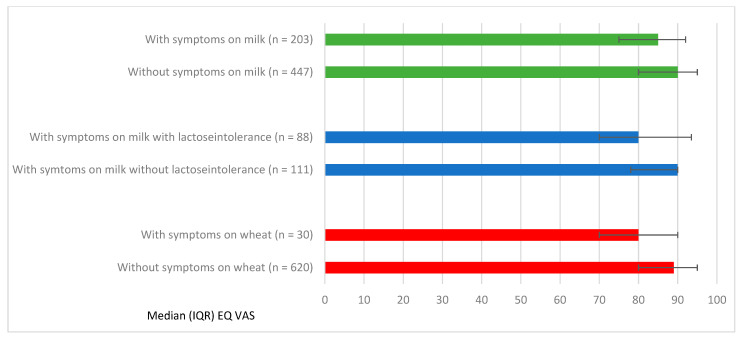
Median EQ VAS and Interquartile Range (IQR) in relation to FHS with specific symptoms to milk and wheat.

Symptoms to milk and wheat were most strongly associated with lower EQ-VAS scores. Those with symptoms to milk (n = 203) had a lower median EQ-VAS compared to without symptoms to milk (n = 447; median 85 vs. 90, *p* = 0.03). This was also presented in the adjusted analyses, with a median EQ-VAS: β-coefficient-5, 95% CI (−7.03, −2.97). A similar pattern was found for wheat (median 80 vs. 89, *p* = 0.04; Figure 2). However, due to the limited sample size, no adjusted analyses could be performed.

In total, 14% (n = 88) of adolescents with FHS reported physician-diagnosed lactose intolerance, a majority reported mild to moderate symptoms (86%) and 14% reported severe symptoms. Adolescents with physician-diagnosed lactose intolerance had a significantly lower EQ-VAS than those without physician-diagnosed lactose intolerance but also reported symptoms for milk (n = 111; median 80 vs. 90 *p* = 0.03; Figure 2). In the adjusted analyses, lactose intolerance was associated with lower median EQ-VAS (β-coefficient-7, 95% CI (−12.05, −1.95) and more problems in the dimension anxiety/depression (OR_adj_ 2.22, 95% CI (1.11–4.42).

### 3.5. Sensitivity Analyses

Adolescent-reported responses to the question about any food reactions the last 12 months in association with the EQ-5D were comparable to parent-reported FHS, except for a stronger impact on EQ-VAS among adolescents’ responses. Those adolescents who reported food reactions had more problems compared to those with no reported food reactions, in the dimensions pain/discomfort (30.25% vs. 16.59%, *p* < 0.001) and anxiety/depression (31.76% vs. 20.99%, *p* < 0.001) and in the EQ-VAS scale (85 vs. 90, *p* < 0.001). A contingency table with subjects of parent-reported FHS compared to adolescents reported food reactions showed an overlap where the number of 370 conforms to both parental FHS and adolescent-reported food reactions (Table 5). Adolescent-reported reactions without corresponding parent reports were reported by 159 participants, and there were 280 parent-reported cases of FHS without corresponding adolescent reports.

## 4. Discussion

This study, based on data from a population-based birth cohort, presents important information of how FHS may impact on self-reported HRQoL in adolescence. In our study, 22% of adolescents experienced FHS symptoms, and FHS was associated with lower HRQoL in adolescence, irrespective of phenotype (IgE-sensitization, allergic co-morbidity, symptom severity). Among those with FHS, females had lower HRQoL than males. Additionally, among those with FHS, symptoms to wheat and milk were associated with lower HRQoL, as was physician-diagnosed lactose intolerance.

### 4.1. Strength and Limitations

The strengths of the present study include the population-based design with the use of self-reported information on HRQoL, the large study sample, and the high response rate. The available data also permitted consideration of different FHS phenotypes, specifically IgE sensitization, severity of symptoms and allergic comorbidity. Additionally, we used a valid and generic HRQoL instrument, EQ-5D, which covers a range of conditions. The instrument has been shown to be valid in illnesses of the skin, respiratory, endocrine, nutritional and metabolic diseases, where FHS may be included [41]. One could argue that a disease specific instrument such as the Food Allergy Quality of Life Questionnaire would have been preferable. However, a disease-specific instrument can only be used among those with the disease, thereby precluding any possibility of comparison to those with or without a specific disease. Whereas a generic instrument such as EQ-5D cannot capture some of the nuances specific to a disease state, it can be used in different populations for comparing HRQoL among those affected and not affected by the disease.

We acknowledge some weaknesses of our study. Our FHS data are not based on oral food challenges, but rather derived from parent-reported food-related symptoms, which we further categorized as mild-to-moderate or severe disease. The definition of milk is based on specific symptoms to milk but may also be a marker for FHS on other dairy products. Further, our definition of lactose intolerance was based on parent-reported physician diagnosis in combination with reported symptoms to milk, yet not necessarily verified with other diagnostic tests, [42], which may be a weakness. We also acknowledge that FHS was based on parent-reported data (aside from IgE sensitization), which may not consistently align with adolescent-reported data. Nonetheless, a sensitivity analysis provided evidence that these types of data were comparable, except for lower EQ-VAS among adolescents with food reactions when using adolescent-reported data.

### 4.2. Impact on HRQoL among Adolescents with FHS

Adolescents with FHS reported lower HRQoL compared to adolescents without FHS, especially in the dimensions of anxiety/depression, and pain/ discomfort and in the EQ-VAS scale. Emotional and behavioral problems, particularly anxiety and depression, among adolescents with FHS, have also been reported by others [43]. In previous studies, adolescents with diagnosed food allergy have reported social isolation as the most distressing aspect of their disease [44,45]. This may influence the mental health of adolescents [46]. Our study extends the findings from these previous reports, by providing evidence that the level of impairment in HRQoL was comparable between adolescents with FHS, regardless of IgE sensitization.

Compared to children with FHS, adolescents with FHS experience a greater reduction in HRQoL [30,47]. Whereas children with FHS are more dependent on their parents, the increased independence associated with adolescence creates additional challenges that may affect their health. Stukus et al. showed that allergic conditions may adversely affect health during this transition phase, given that adolescents are inclined toward risk-taking behaviors [48].

Herein, adolescents with FHS with the most severe symptoms or those with IgE-sensitization were not found to have lower HRQoL. These findings align with those from a Danish study, in which the severity of allergic symptoms did not further contribute to impaired HRQoL [49]. Likewise, Venter et al. showed that HRQoL among 10-year-old children did not differ between those with physician-diagnosed FHS (food allergy or proven with IgE- test) or a perceived, parent-reported FHS [50].

In the present study, females with FHS had a lower HRQoL compared to males with FHS. The same was seen by Wassenberg et al. [51], who noted that girls 0–12 years with FHS in combination with IgE sensitization had lower parent-reported HRQoL than boys with the same condition. Stensgaard et al. reported similar findings but between adults, where females with food allergy had a lower HRQoL than males [49]. We previously showed that age and sex have an impact on HRQoL where young adult males (18–39 years old) had worse food allergy-related HRQoL than adolescent males (<18 years), whereas females with severe symptoms had the lowest HRQoL [52].

When planning for this study, we wanted to minimize the risk that report of food disorders could be a proxy for a personality type who experiences more anxiety and stress and thereby, reports more food-related symptoms. To handle this possible bias, we obtained data from the Swedish Prescribed Drug register of dispended medication to compare if adolescents with and without FHS differed with respect to dispensed antianxiety drugs, antidepressant drugs, and migraine medication as a marker of other symptomatic symptoms, which may explain the differences in HRQoL. However, our findings showed that there were no significant differences in drugs dispensed between the two groups.

### 4.3. The Importance of Correct Diagnoses and Appropriate Care for Adolescents with FHS

In our study population, 22% of adolescents reported FHS symptoms. This high prevalence can be seen as a public health problem, which underscores the need to support young people with FHS symptoms to make cautious and well-informed dietary choices. Young people, including those with FHS, are increasingly turning to social media for health information [42], which may be of variable quality. It would therefore be beneficial for adolescents with FHS to achieve support from healthcare professionals to guide them to approved sources [53,54].

In this context, it is important to highlight the importance of a correct diagnosis: if an individual believes they have a food allergy, their HRQoL may be impaired in multiple ways. A correct diagnosis is essential, to avoid unnecessary dietary restrictions and possible impaired HRQoL. One excellent example of this is The Finnish Allergy Program, where more certified diagnostic testing centers were opened for allergic diseases, which resulted in a 43% decrease in allergy diets (from 7.7% to 4.3%) in primary schools [55]. Although this program was designed for young children, it may also be transferable to an adolescent population.

Further, implementing instruments of HRQoL in clinical practice and not only in research would be beneficial. Assessments of HRQoL may permit identification of a range of problems that can affect adolescents with FHS. Healthcare professionals may communicate these kinds of problems and support the adolescent to understand the consequences of their illness [21].

The observation that adolescents with FHS have impaired HRQoL irrespective of severity, comorbidity or sensitization indicates a need for support from health care professionals with competence in FHS. Healthcare professionals are encouraged to appreciate that impaired HRQoL among those with FHS is seemingly not impacted by IgE sensitization. As such, adolescents with FHS, regardless of IgE status, may benefit from support without minimizing the needed care among those with a more severe FHS who are at risk for fatal allergic reactions [56].

Further studies are needed to understand how adolescents with FHS live and manage their daily life. Underlying factors, including physical, psychological, or social factors, may contribute to the explanation as to why adolescents with FHS have lower HRQoL than their peers without FHS, and thus, warrant further investigation.

## 5. Conclusions

In our population-based birth cohort, FHS is associated with impaired HRQoL in adolescence, irrespective of IgE sensitization, level of symptom severity or allergic comorbidity. Females with FHS had lower HRQoL than males. Symptoms for wheat, milk and physician-diagnosed lactose intolerance among those with FHS were associated with the lowest HRQoL. Healthcare professionals are encouraged to be aware of the impact on HRQoL among adolescents with FHS and ensure a proper diagnosis and support them on how to manage their illness regardless of severity.

## Figures and Tables

**Table 1:** Distribution of EQ-5D in relation to food hypersensitivity (FHS) ^a^.

Dimensions	No FHS n = 2340 (%)	FHS n = 650 (%)	Crude OR ^a^ (95% CI)	Crude *p*-Value ^c,d^	Adjusted ^b,d^ OR (95% CI)	Adjusted ^b,d^ *p*-Value
Mobility			1.64 (0.77–3.49)	0.19	1.29 (0.54–3.13)	0.57
No Problems	2318 (99.06)	640 (98.46)				
Some Problems	21 (0.90)	10 (1.54)				
Extreme Problems	1 (0.04)	0 (0.00)				
Self-Care			0.56 (0.17–1.92)	0.35	0.43 (0.10–1.93)	0.27
No Problems	2321 (99.19)	647 (99.54)				
Some Problems	10 (0.43)	1 (0.15)				
Extreme Problems	9 (0.38)	2 (0.31)				
Usual Activities			1.54 (1.01–2.35)	0.04	1.51 (0.94–2.44)	0.08
No Problems	2264 (96.75)	618 (95.08)				
Some Problems	68 (2.91)	32 (4.92)				
Extreme Problems	8 (0.34)	0 (0.00)				
Pain or Discomfort			1.49 (1.22–1.85)	<0.001	1.37 (1.08–1.72)	0.007
No Problems	1930 (82.48)	493 (75.85)				
Some Problems	402 (17.18)	156 (24.00)				
Extreme Problems	8 (0.34)	1 (0.15)				
Anxiety or Depression			1.31 (1.08–1.60)	0.007	1.24 (0.99–1.55)	0.05
No Problems	1831 (78.25)	476 (73.23)				
Some Problems	479 (20.47)	164 (25.23)				
Extreme Problems	30 (1.28)	10 (1.54)				
EQ VAS			Crude Beta-coefficient (95% CI)	Crude *p*-value	Adjusted ^c^ Beta-coefficient (95% CI)	Adjusted ^c^ *p*-value
EQ VAS Mean (SD)	85.68 (13.31)	83.91 (14.19)		0.003 ^e^	−1.4 (−2.64−0.19)	0.023
	No FHS	FHS	Crude Beta-coefficient (95% CI)		Adjusted ^c^ Beta-coefficient (95% CI)	
EQ VAS Median (IQR)	90 (80–95)	89 (80–94.5)		0.001 ^f^	0 (−0.97−0.98)	1.00

^a^ Food hypersensitivity was defined as parent-reported specific symptom(s) to a specific food(s) in the past 12 months; ^b^ Adjusted for male, socioeconomic status, overweight/obesity and self-reported smoking with logistic-,linear- and quantile regression; ^c^ Differences between groups with regard to the number of subjects with and without reported problems in the EQ-5D descriptive system were tested with chi-square test statistic; ^d^ Some problems and extreme problems in the EQ-5D dimensions were collapsed before conducting the analyses; ^e^ Differences between groups with regard to mean EQ VAS scores were tested with *t*-test; ^f^ Differences between groups with regard to median EQ VAS scores were tested with a two-sample Wilcoxon–Mann–Whitney’s test. FHS—food hypersensitivity; IQR—interquartile range; SD—standard deviation; VAS—Visual Analogue Scale.

**Table 2:** Distribution of EQ-5D among adolescents with food hypersensitivity (FHS) in relation to sex.

Dimensions	Females with FHS n = 363 (%)		Males with FHS n = 287 (%)		Crude OR ^a^ (95% CI)	Crude *p*-Value	Adjusted ^b^ OR ^a^ (95% CI)	Adjusted ^b^ *p*-Value
Mobility					0.84 (0.24–3.01)	0.79	0.86 (0.18–3.98)	0.846
No Problems	357	(98.35)	283	(98.61)				
Some Problems	6	(1.65)	4	(1.39)				
Extreme Problems	0	(0.00)	0	(0.00)				
Self-Care					2.54 (0.23–28.16)	0.45	1.38 (0.08–22.19)	0.821
No Problems	362	(99.72)	285	(99.30)				
Some Problems	0	(0.00)	1	(0.35)				
Extreme Problems	1	(0.28)	1	(0.35)				
Usual Activities					0.75 (0.36–1.56)	0.78	0.79 (0.35–1.79)	0.57
No Problems	343	(94.49)	275	(95.82)				
Some Problems	20	(5.51)	12	(4.18)				
Extreme Problems	0	(0.00)	0	(0.00)				
Pain or Discomfort					0.49 (0.33–0.71)	<0.001	0.51 (0.34–0.77)	0.001
No Problems	255	(70.25)	238	(82.93)				
Some Problems	107	(29.48)	49	(17.07)				
Extreme Problems	1	(0.28)	0	(0.00)				
Anxiety or Depression					0.54 (0.38–0.78)	<0.001	0.55 (0.37–0.82)	0.003
No Problems	247	(68.04)	229	(79.79)				
Some Problems	111	(30.58)	53	(18.47)				
Extreme Problems	5	(1.38)	5	(1.74)				
EQ VAS	Female		Male		Crude Beta-coefficient (95% CI)	Crude *p*-value	Adjusted Beta-coefficient (95%CI)	Adjusted *p*-value
Mean (SD)	82.62 (14.49)		85.53 (13.67)		2.91 (0.72–5.10)	0.009	2.88 (0.64–5.12)	0.012
					Crude Beta-coefficient (95%CI)		Adjusted ^c^ Beta-coefficient (95%CI)	
Median (IQR)	85 (75–90)		90 (80–95)		5.0 (2.31–7.69)	0.002	2.0 (1.24–6.76)	0.005

^a^ Some problems and extreme problems in the EQ-5D dimensions were collapsed before conducting the logistic analysis; ^b^ Adjusted for socioeconomic status, overweight/obesity and self-reported smoking. FHS—food hypersensitivity; IQR—interquartile range; SD—standard deviation; VAS—Visual Analogue Scale.

**Table 3:** Distribution of EQ-5D among adolescents with food hypersensitivity (FHS) in relation to mild/moderate ^a^ and severe ^b^ allergic symptoms.

Dimensions	FHS with Mild-to-Moderate Allergic Symptoms ^a^ n = 438	FHS with Severe Allergic Symptoms ^b^ n = 155	*p*-Value ^c,d^
Mobility			0.62
No Problems	432 (98.63)	152 (98.06)	
Some Problems	6 (1.37)	3 (1.94)	
Extreme Problems	0 (0.00)	0 (0.00)	
Self-Care			0.30
No Problems	435 (99.32)	155 (100)	
Some Problems	0 (0.00)	0 (0.00)	
Extreme Problems	1 (0.00)	0 (0.00)	
Usual Activities			0.89
No Problems	417 (95.21)	148 (95.48)	
Some Problems	21 (4.79)	7 (4.52)	
Extreme Problems	0 (0.00)	0 (0.00)	
Pain or Discomfort			0.64
No Problems	331 (75.57)	120 (77.42)	
Some Problems	106 (24.20)	35 (22.58)	
Extreme Problem	1 (0.23)	0 (0.00)	
Anxiety or Depression			0.39
No Problems	321 (73.29)	119 (76.77)	
Some Problems	108 (24.66)	36 (23.23)	
Extreme Problems	9 (2.05)	0 (0.00)	
EQ VAS Mean (SD)	84.0 (14.5)	83.8 (11.9)	0.87 ^e^
EQ VAS Median (IQR)	89.5 (80–95)	86 (80–90)	0.27 ^f^

^a^ Mild to moderate symptoms define as separate symptoms of itchy nose, stuffy nose, runny nose, itchy eyes, nettle rash that covered less, stomach pain/vomiting, swollen in face, eyelids, lips, itch in mouth throat ears; ^b^ Severe symptoms define as separate symptoms of: difficulty breathing asthma cough, nettle rash covering most of the body, hoarseness, indistinct speech, pronounced fatigue, decreased awareness, unconsciousness or mild to moderate symptoms together with severe symptoms; ^c^ Some problems and extreme problems in the EQ-5D dimensions were collapsed before conducting the analysis, ^d^ differences between groups with regard to the number of subjects with and without reported problems in the EQ-5D descriptive system were tested with chi-square test statistic. ^e^ Differences between groups with regard to mean EQ-VAS scores were tested with *t*-test; ^f^ Differences between groups with regard to median EQ-VAS scores were tested with a two sample Wilcoxon–Mann–Whitney’s rank-sum test. FHS—Food hypersensitivity; IQR—interquartile range; SD—standard deviation; VAS—Visual Analogue Scale.

**Table 4:** Distribution of EQ-5D among adolescents with food hypersensitivity (FHS) in relation to IgE-sensitization (fx5 ^®^).

Dimensions	FHS with IgE Sensitization n = 164 (%)	FHS without IgE Sensitization n = 397 (%)	*p*-Value ^a,b^
Mobility			0.10
No Problems	160 (97.56)	394 (99.24)	
Some Problems	4 (2.44)	3 (0.76)	
Extreme Problems	0 (0.00)	0 (0.00)	
Self-Care			0.52
No Problems	164 (100.00)	396 (99.75)	
Some Problems	0 (0.00)	1 (0.25)	
Extreme Problems	1 (0.28)	0 (0.00)	
Usual Activities			0.70
No Problems	157 (95.73)	377 (94.96)	
Some Problems	7 (4.27)	20 (5.04)	
Extreme Problems	0 (0.00)	0 (0.00)	
Pain or Discomfort			0.81
No Problems	126 (76.83)	303 (76.32)	
Some Problems	38 (23.17)	93 (23.43)	
Extreme Problems	0 (0.00)	1 (0.25)	
Anxiety or Depression			0.65
No Problems	117 (71.34)	297 (74.81)	
Some Problems	44 (26.83)	95 (23.93)	
Extreme Problems	3 (1.83)	5 (1.26)	
EQ VAS Mean (SD)	84.47(12.91)	83.95 (13.9)	0.68 ^c^
EQ VAS Median (IQR)	88 (80–90)	87 (80–95)	0.75 ^d^

^a^ Some problems and extreme problems in the EQ-5D dimensions were collapsed before conducting the analysis; ^b^ Differences between groups with regard the number of subjects with and without reported problems in the EQ-5D descriptive system were tested with chi-square test statistic; ^c^ Differences between groups with regard to mean EQ VAS scores were tested with t-test; ^d^ Differences between groups with regard to median EQ VAS scores were tested with a two sample Wilcoxon–Mann–Whitney’s rank-sum test. FHS—food hypersensitivity; IgE—Immunoglobulin E; IQR—interquartile range; SD—standard deviation; VAS—Visual Analogue Scale.

**Table 5:** Parent-reported food hypersensitivity (FHS) vs. adolescent-reported food reactions.

	Parent-Reported FHS ^a^	
Adolescent-Reported Food Reactions ^b^	No	Yes
No	2174	280
Yes	159	370

^a^ Food hypersensitivity was defined as specific parent-reported symptom(s) to a specific food(s) in the past 12 months; ^b^ ‘Food reactions’ was defined as adolescents reporting any symptoms to foods the last 12 months.

## Data Availability

The datasets generated and/or analyzed during the current study are not publicly available since the dataset contains sensitive personal data but are available from the corresponding author on reasonable request.

## Supplementary Materials

The following are available online at https://www.mdpi.com/article/10.3390/nu13072357/s1, Table S1: Distribution of baseline characteristics of the entire cohort (n = 4089) and the study population (n = 2990), Table S2: Distribution of EQ 5D in the study-population in relation to selected background characteristics, Table S3: Distribution of food hypersensitivity with and without allergic comorbidity (asthma and/or eczema and/or, rhinitis), Table S4: Distribution of EQ -5D in relation to food hypersensitivity and symptoms on common foods, nuts/peanuts and other specific foods and Text S1 Definitions: Allergic comorbidity (FHS in combination with asthma, eczema or rhinitis).

## Abbreviations

BAMSE	Swedish abbreviation for Children, Allergy, Milieu, Stockholm, Epidemiology
FHS	Food hypersensitivity
HRQoL	Health Related Quality of Life
IgE sensitization	Immunoglobulin E sensitization
