# Supplementary material for: Living with Food Hypersensitivity as an Adolescent Impairs Health Related Quality of Life Irrespective of Disease Severity: Results from a Population-Based Birth Cohort
Source: Nutrients. 2021 Jul 9;13(7):2357. doi: 10.3390/nu13072357 (PMC8308910; doi:10.3390/nu13072357)
Supplement: Supplementary file 1 [file nutrients-13-02357-s001.zip › nutrients-1210543-supplementary.pdf]

**Text S1**

**Definitions**

**Allergic comorbidity (FHS in combination with asthma, eczema or rhinitis)**

**Asthma**

More than 3 episodes of wheeze in the last 12 months prior to the date of 16-year follow-up questionnaire or at least 1 episode of wheeze in the last 12 months prior to the date of 16-year follow-up questionnaire, in combination with prescribed inhaled steroids occasionally or regularly. (parent-reported)

**Eczema**

Dry skin in combination with itchy rash and typical localisation (arms/legs flexures or wrists/ankles or neck) in the last 12 months prior to date of 16-year follow-up questionnaire. (parent-reported)

**Rhinitis**

Symptoms of sneezing, a runny or blocked nose, or itchy, red and watery eyes after exposure to furred pets or pollen in the last 12 months prior to the date of 16-year follow-up questionnaire. (parent-reported)

**Other health covariates**

**Overweight/obesity**

Defined per gender-specific and age-specific cut-off values proposed by the International Obesity Task Force (1).

**Socioeconomic status**

Defined as socioeconomic status at birth for the household according to dominance order in 2 classes (low: blue collar vs high white collar) according to the Nordic standard occupational classification.

**Table S1: Distribution of baseline characteristics of the entire cohort (N=4089) and the study population (n=2990)**

| Background factors | Cohort (N=4,089) | Study population (n=2990) | |
|---|---|---|---|
| | n (% ) | n ( % ) | 95% CI |
| **Male sex** | 2065 ( 50.5) | 1204 (49.1) | 48.2-50.1 |
| **Parental allergy[a]** | 1200 ( 29.7) | 908 (30.6) | 29.7.0-31.5 |
| **Young mother(<25 years)** | 319 (7.8 ) | 215 (7.2) | 6.7-7.7 |
| **Either parent smoked at baseline** | 783 (21.0) | 594 (20.0) | 19.2-20.8 |
| **Parental white collar workers[b]** | 3323 (82.7 ) | 2481 (84.2) | 83.5-85.0 |

[a] Heredity: mother and/or father with doctor-diagnosed asthma and asthma medication and/or doctor-diagnosed hay fever in combination with furred pets- and/or pollen allergy.
[b]White collar workers; defined according to the Nordic standard occupational classification and Swedish socio-economic classification

## Table S2: Distribution of EQ 5D in the study-population in relation to selected background characteristics

| Dimensions | Study population | Male | | Allergic heredity | | Socioeconomic status; Parental white collar workers | | Smoking at 16 years | | Overweight/obesity at 16 year | |
|---|---|---|---|---|---|---|---|---|---|---|---|
| | | Yes | No | Yes | No | Yes | No | Yes | No | Yes | No |
| | n= 2990 | n=1470 (%) | n=1520(%) | n= 908 (%) | n=2055(%) | n=2481(%) | n=465 (%) | N=353 (%) | n= 2632 (%) | 427 (%) | 2099 (%) |
| Mobility | | | | | | | | | | | |
| **No problems** | 2958(99.93) | 1458 (99.18) | 1500 (98.68) | 897 (99.03) | 2035 (99.03) | 2457 (98.28) | 457 (98.28) | 346 (98.02) | 2607 (99.05) | 417 (97.66) | 2084 (99.29) |
| **Some problems** | 31 (1.04) | 12 (0.82) | 19 (1.25) | 11 (1.21) | 19 (0.92) | 24 (0.97) | 7 (1.51) | 7 (1.98) | 24 (0.91) | 10 (2.34) | 14 (0.67) |
| **Extreme problems** | 1 (0.03) | 0 (0.00) | 1 (0.07) | 0 (0.00) | 1 (0.05) | 0 (0.00) | 1 (0.22) | 0 (0.00) | 1 (0.04) | 0 (0.00) | 1 (0.05) |
| **p-value** [a,b] | | 0.18 | | 0.56 | | 0.15 | | 0.165 | | 0.005 | |
| Self-care | | | | | | | | | | | |
| **No problems** | 2968 (99.26) | 1462 (99.46) | 1506 (99.08) | 902 (99.34) | 2039 (99.22) | 2463 (99.27) | 461 (99.14) | 350 (99.15) | 2613 (99.28) | 424 (99.30) | 2085 (99.33) |
| **Some problems** | 11 (0.37) | 4 (0.27) | 7 (0.46) | 1 (0.11) | 10 (0.49) | 8 (0.32) | 3 (0.65) | 0 (0.00) | 11 (0.42) | 2 (0.47) | 5 (0.24) |
| **Extreme problems** | 11 (0.37) | 4 (0.27) | 7( 0.46) | 5 (0.55) | 6 (0.29) | 10 (0.40) | 1 (0.22) | 3 (0.85) | 8 (0.30) | 1 (0.23) | 9 (0.43) |
| **p-value** [a,b] | | 0.23 | | 0.73 | | 0.76 | | 0.79 | | 0.93 | |
| Usual activities | | | | | | | | | | | |
| **No problems** | 2264 (96.75) | 1422 (96.73) | 1460 (96.05) | 873 (96.15) | 1984 (96.55) | 2399 (96.69) | 442 (95.05) | 325 (92.07) | 2552 (96.96) | 412 (96.49) | 2025 (96.47) |
| **Some problems** | 68 (2.91) | 45 (3.06) | 55 (3.62) | 31 (3.41) | 67 (3.26) | 74 (2.98) | 23 (4.95) | 28 (7.93) | 72 (2.71) | 14 (3.28) | 69 (3.29) |
| **Extreme problems** | 8 (0.27) | 3 (0.20) | 5 (0.33) | 4 (0.44) | 4 (0.19) | 8 (0.32) | 0 (0.00) | 0 (0.00) | 8 (0.30) | 1 (0.23) | 5 (0.24) |
| **p-value** [a,b] | | 0.32 | | 0.59 | | 0.08 | | <0.001 | | 0.99 | |
| Pain or discomfort | | | | | | | | | | | |
| **No problems** | 2423 (81.04) | 1248 (84.90) | 1175 (77.30) | 737 (81.17) | 1671 (81.31) | 2031 (81.86) | 359(77.20) | 271 (76.77) | 2148 (81.61) | 324 (75.88) | 1714 (81.66) |
| **Some problems** | 558 (18.66) | 219 (14.90) | 339 (22.30) | 169 (18.61) | 377 (18.35) | 442 (17.82) | 105(22.58) | 82 (23.23) | 475 (18.05) | 100 (23.42) | 380 (18.0) |
| **Extreme problems** | 9 (0.30) | 3 (0.20) | 6 (0.39) | 2 (0.22) | 7 (0.34) | 8 (0.32) | 1 (0.22) | 0 (0.00) | 9 (0.34) | 3 (0.70) | 5 (0.24) |
| **p-value**[a,b] | | <0.001 | | 0.93 | | 0.02 | | 0.03 | | 0.006 | |
| Anxiety or depression | | | | | | | | | | | |
| **No problems** | 2307 (77.16) | 1243 (84.56) | 1064 (70.00) | 704 (77.53) | 1584 (77.08) | 1921 (77.43) | 351 (75.48) | 233 (66.01) | 2070 (78.65) | 326 (76.35) | 1623 (77.32) |
| **Some problems** | 643 (21.51) | 216 (14.69) | 427 (28.09) | 192 (21.15) | 444 (21.61) | 527 (21.24) | 108 (23.23) | 111 (31.44) | 531 (20.17) | 91 (21.31) | 454 (21.63) |
| **Extreme problems** | 40 (1.34) | 11 (0.75) | 29 (1.91) | 12 (1.32) | 27 (1.31) | 33 (1.33) | 6 (1.29) | 9 (2.55) | 31 (1.18) | 10 (2.34) | 22 (4.05) |
| **p-value** [a,b] | | <0.001 | | 0.79 | | 0.36 | | <0.001 | | 0.66 | |
| EQ VAS **Mean (SD)** | 85.30(13.52) | 86.92 (12.74) | 83.74 (14.07) | 85.63 (13.53) | 85.29 (13.37) | 85.36 (13.50) | 84.99 (13.86 | 80.79(14.70) | 85.91 (13.25) | 84.85 (13.17) | 85.42 (13.20) |
| **p-value**[c] | | <0.001 | | 0.52 | | 0.60 | | <0.001 | | 0.41 | |
| EQ VAS **Median(IQR)** | 90 (80-95) | 90 (80-95) | 86 (80-95) | 90 (80-95) | 90 (80-95) | 90 (80-95) | 90 (80-95) | 85 (75-90) | 90 (80-95) | 90 (80-95) | 90 (80-95) |
| **p-value**[d] | | <0.001 | | 0.33 | | 0.76 | | <0.001 | | 0.23 | |

[a] Some problems and extreme problems in the EQ-5D dimensions were collapsed before conducting the analysis, [d] differences between groups with food symptoms regard to the number of subjects with and without reported problems in the EQ-5D descriptive system and no hypersensitivity were tested with chi-square test statistic. [c] Differences between groups with regard to mean EQ VAS scores were tested with t-test. [d] Differences between groups with regard to median EQ VAS scores were tested with a two sample Wilcoxon-Mann-Whitney test and p-value of ≤ 0.05 was considered significant. Socioeconomic groups according to Statistics Sweden

**Table S3: Distribution of food hypersensitivity with and without allergic comorbidity (asthma and/or eczema and/or, rhinitis)**

| Dimensions | **Food hypersensitivity with allergic comorbidity (asthma, eczema, or rhinitis) n=379** | **Food hypersensitivity without allergic comorbidity (asthma, eczema or rhinitis) n=256** | **p-value [a,b]** |
|---|---|---|---|
| Mobility | | | |
| **No problems** | 375 (98.94) | 250 (98.56) | |
| **Some problems** | 4 (1.06) | 6 (1.44) | |
| **Extreme problems** | 0 (0.00) | 0 (0.00) | 0.20 |
| Self-care | | | |
| **No problems** | 377 (99.47) | 255 (99.61) | |
| **Some problems** | 1 (0.26) | 1 (0.39) | |
| **Extreme problems** | 1 (0.26) | 0 (0.00) | 0.80 |
| Usual activities | | | |
| **No problems** | 362 (95.51) | 241 (94.14) | |
| **Some problems** | 17 (4.49) | 15 (5.86) | 0.44 |
| **Extreme problems** | 0 (0.00) | 0 (0.00) | |
| Pain or discomfort | | | |
| **No problems** | 293 (77.31) | 186 (72.66) | |
| **Some problems** | 85 (22.43) | 70 (27.34) | |
| **Extreme problems** | 1 (0.26) | 0 (0.00) | 0.18 |
| Anxiety or depression | | | |
| **No problems** | 288 (75.99) | 177 (69.14) | |
| **Some problems** | 86 (22.69) | 74 (28.91) | 0.06 |
| **Extreme problems** | 5 (1.32) | 5 (1.95) | |
| EQ VAS **Mean (SD)** | 84.54 (12.76) | 88 (80-92) | 0.17 [c] |
| EQ VAS **Median (IQR)** | 83.01 (15.41) | 89.5 (75-95) | 0.65 [d] |

[a] Some problems and extreme problems in the EQ-5D dimensions were collapsed before conducting the analysis, [b]differences between groups with regard to the number of subjects with and without reported problems in the EQ-5D descriptive system were tested with chi-square test statistic. [c] Differences between groups with regard to mean EQ VAS scores were tested with t-test [d]Differences between groups with regard to median EQ VAS scores were tested with a two sample Wilcoxon-Mann-Whitney test and p-value of ≤ 0.05 was considered significant.

**Table S4: Distribution of EQ 5D in relation to food hypersensitivity and symptoms to common foods [a] , nuts/peanut [b] and other specific foods [c]**

| Dimensions | Food hypersensitivity with symptoms on common foods* n= 250 (%) | Food hypersensitivity with no symptoms on common food n=400 (%) | Food hypersensitivity with symptoms on nuts and peanuts n=270 (%) | Food hypersensitivity with no symptoms on nuts and peanuts n=380 (%) | Food hypersensitivity with symptoms on other specific foods n=366 (%) | Food Hypersensitivity with no symptoms on other specific foods n= 284 (%) |
|---|---|---|---|---|---|---|
| Mobility | | | | | | |
| **No problems** | 245 (98.00) | 395 (98.75) | 267 (98.89) | 373 (98.16) | 361 (98.63) | 279 (98.24) |
| **Some problems** | 5 (2.00) | 5 (1.25) | 3 (1.11) | 7 (1.84) | 5 (1.37) | 5 (1.76) |
| **Extreme problems** | 0 (0.00) | 0 (0.00) | 0 (0.00) | 0 (0.00) | 0 (0.00) | 0 (0.00) |
| ***p*-value [d,e]** | 0.45 | | 0.46 | | 0.69 | |
| Self-care | | | | | | |
| **No problems** | 250 (100) | 397 (99.25) | 268 (99.26) | 379 (99.74) | 364 (99.45) | 283 (99.65) |
| **Some problems** | 0 (0.00) | 1 (0.25) | 1 (0.37) | 0 (0.00) | 1 (0.27) | 0 (0.00) |
| **Extreme problems** | 0 (0.00) | 2 (0.50) | 1 (0.37) | 1 (0.26) | 1 (0.27) | 1 (0.35) |
| **p-value[d,e]** | 0.17 | | 0.48 | | 0.72 | |
| Usual activities | | | | | | |
| **No problems** | 238 (95.20) | 380 (95.0) | 259 (95.93) | 359 (94.47) | 348 (95.08) | 270 (71.65) |
| **Some problems** | 12 (4.80) | 20 (5.0) | 11(4.07) | 21 (5.53) | 18 (4.92) | 14 (4.93) |
| **Extreme problems** | 0 (0.00) | 0 (0.00) | 0 (0.00) | 0 (0.00) | 0 (0.00) | 0 (0.00) |
| **p-value[d,e]** | 0.90 | | 0.40 | | 0.99 | |
| Pain or discomfort | | | | | | |
| **No problems** | 182 (72.80) | 311 (77.75) | 210 (77.78) | 283 (74.47) | 281 (76.78) | 212 (71.65) |
| **Some problems** | 68 (27.20) | 88 (22.0) | 59 (21.85) | 97 (25.53) | 84 (22.95) | 72 (25.35) |
| **Extreme problems** | 0 (0.00) | 1 (0.25) | 1 (0.37) | 0 (0.00) | 1 (0.27) | 0 (0.00) |
| **p-value [d,e]** | 0.15 | | 0.28 | | 0.53 | |
| Anxiety or depression | | | | | | |
| **No problems** | 183 (73.20) | 293 (73.25) | 203 (75.19 | 273 (71.84) | 274 (74.86) | 202 (71.13 |
| **Some problems** | 64 (25.60) | 100 (25.0) | 64 (23.70) | 100 (26.32) | 85 (23.22) | 79 (27.82) |
| **Extreme problems** | 3 (1.20) | 7 (1.75) | 3 (1.11) | 7 (1.84) | 7 (1.91) | 3 (1.06) |
| **p-value [d,e]** | 0.99 | | 0.25 | | 0.29 | |
| EQ VAS **Mean (SD)** | 81.97 (16.17) | 85.12 (12.68) | 84.80 (13.11) | 83.28 (14.90) | 84.22 (14.77) | 83.5 (13.43) |
| **p-value [f]** | 0.06 | | 0.18 | | 0.52 | |
| EQ VAS **Median (IQR)** | 85 (75-95) | 90 (80-95) | 88.5 (80-92) | 87.5 (75-95) | 90 (80-95) | 86 (79.5-91) |
| **p-value [g]** | 0.04 | | 0.12 | | 0.21 | |

[a]Symptoms to common foods defined as specific symptoms the last 12 months, or avoidance because of previous reactions or results of allergy testing on milk and/or egg and/or fish and/or wheat.

b Symptoms to nuts and peanuts defined as specific symptoms the last 12 months, or avoidance because of previous reactions or results of allergy testing on peanut and/or hazelnut and/or almond and/or walnut, pecan and/or cashew/pistachio and/or Brazil nut

[c]Symptoms to other specific foods defined as specific symptoms the last 12 months, or avoidance because of previous reactions or results of allergy testing shellfish and/ or, soy and/or sesame seed and or apple/pear and/or peach/nectarine/ plum/cherry and/or kiwi and or banana and/or raw carrot.

[d]Some problems and extreme problems in the EQ-5D dimensions were collapsed before conducting the analysis.

[e]Differences between groups with food symptoms regard to the number of subject with and without reported problems in the EQ-5D descriptive system and no hypersensitivity were tested with chi-square test.

[f] Differences between groups with regard to mean EQ VAS scores were tested with t-test. [g] Differences between groups with regard to median EQ VAS scores were tested with a two sample Wilcoxon-Mann-Whitney test and p-value of ≤ 0.05 was considered significant.

## References

1. Cole TJ, Lobstein T. Extended international (IOTF) body mass index cut-offs for thinness, overweight and obesity. Pediatr Obes. 2012;7(4):284-94.
